# Self-determination theory in health care and its relations to motivational interviewing: a few comments

**DOI:** 10.1186/1479-5868-9-24

**Published:** 2012-03-02

**Authors:** Edward L Deci, Richard M Ryan

**Affiliations:** 1 Department of Clinical & Social Sciences in Psychology, University of Rochester, PO Box 270266, Rochester. NY 14627, USA

## Abstract

The papers of this special issue have the dual focus of reviewing research, especially clinical trials, testing self-determination theory (SDT) and of discussing the relations between SDT and motivational interviewing (MI). Notably, trials are reviewed that examined interventions either for behaviors such as physical activity and smoking cessation, or for outcomes such as weight loss. Although interventions were based on and intended to test the SDT health-behavior-change model, authors also pointed out that they drew techniques from MI in developing the interventions. The current paper refers to these studies and also clarifies the meaning of autonomy, which is central to SDT and has been shown to be important for effective change. We clarify that the dimension of autonomy versus control is conceptually orthogonal to the dimension of independence versus dependence, and we emphasize that autonomy or volition, not independence, is the important antecedent of effective change. Finally, we point out that SDT and MI have had much in common for each has emphasized autonomy. However, a recent MI article seems to have changed MI's emphasis from autonomy to change talk as the key ingredient for change. We suggest that change talk is likely to be an element of effective change only to the degree that the change talk is autonomously enacted and that practitioners facilitate change talk in an autonomy supportive way.

## 

With all the resources and energy being spent on biological aspects of health care, it remains critical to gain a better understanding of its behavioral components-from the promotion of healthy lifestyles to the facilitation of adherence to medical advice. Behavioral inputs account for considerable variance in health outcomes and provide a ready focus for intervention.

This excellent collection of papers represents an important step in addressing the health-relevant changes of increasing physical activity, improving diet, fostering smoking cessation, and facilitating weight loss-changes that are important for healthier cardiovascular functioning and cancer prevention, among other outcomes. The papers discuss research on health care using concepts, measures, and interventions that have been derived from self-determination theory (SDT) as well as techniques derived from the Motivational Interviewing model (MI).

Although research applying SDT to health care began more than 15 years ago, the quantity of work has greatly increased during this century. Particularly noteworthy has been a group of randomized clinical trials that have begun to appear in the literature, several of which are reviewed and discussed in the papers of this special issue. They are important studies both because they provide support for the SDT approach to patient care and because they lay the groundwork for future, more-refined studies such as ones addressing specific social-contextual factors that promote maintained health-behavior change and improved health.

Central to SDT is the concept of autonomous self-regulation comprising both intrinsic motivation and well-internalized extrinsic motivation. According to SDT, being autonomous refers to acting with a sense of volition and the experience of willingness. As philosophers from Frankfurt [1] to Freidman [2] have highlighted, when one acts autonomously the course of one's action is (or would be) reflectively self-endorsed. Although the issue of volition was long ignored within the literature of behavior change, its importance has become increasingly clear. When people are autonomously motivated, they are more wholeheartedly engaged, persistent, and efficacious than when controlled in their motivations [3].

As pointed out by Vansteenkiste, Williams, and Resnicow [4], autonomy within SDT does not mean acting independently. Indeed, SDT makes two critical distinctions, which are often confused within literatures of development, culture, and behavior change: between being controlled and being dependent, and also between being autonomous and being independent. Within SDT dependence is defined in terms of reliance-one is dependent if one relies on others for goods or guidance. Given this definition, people can reflectively choose to rely on others, thus being autonomously dependent, or they can thoughtfully choose not to be dependent, thus being autonomously independent. Similarly, people can also feel controlled or pressured to depend upon another's lead, a circumstance not rare in health care, because people often feel either the authoritarian pressure of medical care or, sometimes, the introjected voice that they should be compliant. Finally, people can be pushed into independence, as when others force them to "go it alone" or abandon their need for direction. In sum, control and dependence are not the same thing, nor are autonomy and independence.

As such, the opposite of autonomy is not dependence or interdependence as some theories have suggested. Instead, the opposite of autonomy is being heteronomous or controlled. This means being pressured to think, feel, or behave in particular ways whether through coercion or seduction. In our SDT perspective the dimensions of autonomy versus control and independence versus independence are theoretically orthogonal, and in practice it is critical to distinguish them. Much of the discussion in the articles on which we are commenting relates to these issues as they deal with the complexities of both helping and advising while respecting and fostering autonomy.

In health care, as in human development, this distinction between autonomy-as-volition (the SDT view) and autonomy-as-independence (the view of some other theories) is extremely important (see e.g., Soenens, Vansteenkiste, Lens, Luyckx, Goossens, Beyers, & Ryan, [5]), because promoting autonomy among patients, which is advocated within biomedical ethics, does not refer to merely leaving them alone to decide and act for themselves. Rather, it means encouraging them to make choices about how to behave, providing them with the information they need for making the choices, and respecting the choices they make. Particularly with regard to the technical aspects of medicine it means helping by translating information and scaffolding the presentation of facts to facilitate more reflective lifestyle choices and commitments to health-related change, a process in which both reliance on providers and support for autonomy are involved.

### The Influence of Social Environments

Social environments, or specific factors within social environments, that are referred to as autonomy supportive have been found to promote autonomous self-regulation both by helping people maintain intrinsic motivation and facilitating internalization of extrinsic motivation. One person being autonomy supportive of another involves the first person (often an authority figure) accepting and acknowledging the internal frame of reference of the other. The first person further conveys respect for the other, encourages exploration and choice, supports the other's decisions, and refrains from pressuring and controlling the other even in subtle ways such as with contingent regard [6]. The opposite of being autonomy supportive is being controlling. This means using demands and contingencies to pressure people to behave in particular ways. When social environments are autonomy supportive people within them are likely to be more autonomous, and when social environments are controlling people tend to be more controlled.

Within SDT, we emphasize the importance of not being controlling with patients. This means respecting their frame of reference and helping them to chart a pathway of engagement in their own care that they can both endorse and apply. We hasten to add, however, that support of autonomy is not an implicit endorsement of being permissive or neglectful-of encouraging patients to do whatever they want. As experts in their field, health-care practitioners may need to provide structure for their patients, which involve ensuring that patients have relevant information about health risks and about the relations between their behaviors and the consequences likely to be associated with them. It is important, for example, if patients do not understand the empirically validated relations between, say, tobacco use and cardiovascular events, to provide them with that information in a somewhat dispassionate way, or if they do not want to know the risks, to take interest in *that *barrier to change, and what might motivate it. Clearly, this does not mean trying to scare them with the information or using the information to pressure them to not use tobacco. What it does mean is providing them relevant information to use in making their own informed choices about how to behave. Stated differently, it is important when providing patients with structure to be autonomy supportive in doing so, respecting both their ability to make decisions and their desire to be healthy.

### Basic Psychological Needs and the Social Environment

SDT maintains that all human beings have three basic psychological needs that must be satisfied for them to function optimally. These are the needs for competence, autonomy, and relatedness to others. It is important to note that this basic-needs proposition was not formulated from clinical observations or philosophical assumptions, but was formulated empirically while studying the conditions under which people tend to thrive. We began with specific questions such as, what happens to people's intrinsic motivation when they are rewarded for doing an intrinsically interesting activity, and we then gathered data to answer the questions. When the data highlighted reliable phenomena we proposed psychological processes to account for the phenomena and we then tested the proposed processes in further studies. The idea of universal psychological needs was proposed because it seemed like the best way to explain phenomena related to social contextual factors having specific effects on intrinsic motivation and the internalization of extrinsic motivation. After suggesting the idea of basic needs and testing it in multiple ways, we proposed a central SDT proposition: Social-contextual factors that support satisfaction of the three basic psychological needs will promote autonomous functioning, persistence, effective performance (especially on heuristic tasks), and wellness, whereas social-contextual factors that thwart satisfaction of these three basic psychological needs will result in diminished autonomy, poorer performance, less persistence, and greater ill-being. Many studies have confirmed this proposition and have shown its relevance across gender, age, and socioeconomic status, as well as in eastern-collectivist, as well as western-individualistic, cultures.

The concept of autonomy support refers to providing interpersonal conditions that support the person's initiative, volition, and integrity. In other words, it facilitates satisfaction of the basic psychological need for autonomy. As it turns out, autonomy-support has also been found to relate to satisfaction of the other two basic psychological needs. In part this is likely due to the fact that people who support others' autonomy tend also to support their needs for competence and relatedness. But there is likely more to it than that. When one person supports another's autonomy, the other will likely feel greater freedom to behave in ways that result in satisfaction for his or her competence and relatedness needs. So, supporting autonomy typically helps others get all their basic psychological needs satisfied.

In most of the clinical trials testing SDT the foundational role of autonomy support has been confirmed (see Ryan et al. [3] for a brief review). In these interventions practitioners' support for autonomy enhanced patient volition, which is in turn related to greater feelings of efficacy and connection with caregivers. These satisfactions support enduring change.

### Being Autonomy Supportive and Offering Advice

As mentioned in various of the papers (e.g., Patrick & Williams, [7]), SDT health-care interventions can include having health-care professionals provide patients with advice or recommendations, so long as it is done in an autonomy-supportive way. This can be a very tricky business because it is all-too-easy for patients to view health-care professionals as authorities and to interpret their words as controlling. It is thus essential for health-care providers who are giving advice or making recommendations to take pains not to be controlling and to provide the information in a way that is truly an input to patients' choices and not a subtle form of control. For example, practitioners might say to their patients who smoke cigarettes: "As your provider, I would like to advise you that it is important for your health to try giving up smoking cigarettes. I understand that using tobacco can help you feel better at times and that breaking the tobacco habit can be very difficult. I also believe that whether or not you do try to stop is wholly your choice. I merely want to say that I think it would be useful for you to give this serious consideration and to make a choice about whether to continue smoking or to try stopping. I would respect whichever option you chose."

Providers who make statements such as these would be telling their patients what they believe is best for the patients' health, but they would also be acknowledging that the decision is a difficult one and that they, the providers, would respect the patients' decisions. The tone of voice that the practitioners use and the emotions that might be conveyed along with the words would, of course, make a big difference to the effects of the words. They can alter what we call the "functional significance" or meaning of the words, conveying either control or autonomy support, and connectedness or disdain. So it is important for providers to be speaking from a place within themselves where their words are authentic because they-the providers-do truly believe that it is up to the patients to make choices and would genuinely accept the patients' decisions. From the perspective of SDT, providing advice would not be directive or controlling if it were done in a truly autonomy-supportive way.

*Being directive with some patients? *Resnicow and McMaster [8] raised the question of whether it is appropriate for practitioners to be more directive with some patients-to give them relevant information, tell them what to do, and expect them to do it. The authors cited a study of rural African American women [9] and their own experiences treating Mexican Americans with diabetes to argue that patients in these subgroups indicated that they preferred a directive approach in which the doctors gave more advice and told the patients what to do rather than a patient-centered approach in which the doctors gave little information and asked the patients what they wanted to do.

These comments prompt several important points. First, within any subgroup of patients there will be substantial variability in the degree to which patients desire directives from professionals, so it is important not to think in terms of providing a directive approach to subgroups of patients such as Africian-American women or Mexican Americans. Instead, what is important is for practitioners to be able to take each patient's perspective and, to a significant degree, be responsive to the patient's feelings and desires. Practitioners will find that, with some patients regardless of their subgroup, providing more information and making recommendations is useful, whereas other patients will do well with less structure. For those patients who need more, the responsive practitioners will provide more information and recommendations because they believe that doing so would be helpful for the patients. As such, their behaviors would be in accord with the patients' perspectives. Hence, expressing recommendations would not mean that the practitioners were being controlling of the patients, or even really directive. Rather, the practitioners would be providing structure that patients themselves are indicating would be helpful and doing so in an autonomy supportive way. By responding to the patients' internal frame of reference-that is, to their implicit or expressed wishes-the practitioners would be creating the conditons that would promote internalization of recommendations and thus long-term maintenance of healthy behaviors.

As has been stated in several of the papers in this issue, health-relevant changes are likely to endure when patients have internalized and accepted as their own the practitioners' recommendations. This has been shown to occur more reliably when the practitioners are autonomy supportive. That means that the practitioners would be conveying respect for the individual patients by accepting their perspectives, and the practitioners would be encouraging the patients to make their own choices about behaviors related to health risks or chronic conditions. That said, it is worth noting that it may be useful for clinicians to be sensitive to the fact, especially if they only occasionally work with a particular subgroup of patients, that, relative to other subgroups, a higher percentage of this subgroup of patients may require more information and advice. That does not mean, however, "telling them what to do and expecting them to do it," for that is spilling over from autonomy-supportive structure into control, and we know of no evidence supporting the efficacy of controlling interactions.

## Motivational Interviewing and SDT

Motivational Interviewing (MI) is a clinical approach that began as a treatment for addictions and has spread to a broader set of health-relevant behaviors [10]. Implicit in it has been a sensibility derived from Rogers's [11] person-centered approach to counseling which emphasizes that being responsive to and acknowledging patients' feelings is important for their growth and wellness. In line with this sensibility, as argued by Resnicow and McMaster [8], promoting patients' autonomy or volition is important within MI. As such, there is a similar interpersonal perspective to treating people within MI and SDT, and the intervention techniques used within these two approaches also have much in common. Indeed, investigators performing the clinical trials that have been discussed in this issue and were intended to test the SDT model of health-behavior change have indicated that they drew from MI for specific elements of their interventions (e.g., Fortier, Duda, Guerin, & Teixeira, [12]). In doing so, they were acknowledging similarities between the philosophies and aims of MI and SDT.

For example, both approaches can be thought of as being person-centered; both are non-judgmental and supportive; both provide information that is responsive to what the patients' appear to need; both buttress patients' attempts to come into deeper contact with their inner experiences and motivations; and both have emphasized patients' autonomy while at the same time working to promote patients taking responsibility for behaving in healthy ways. Further, as argued by Markland, Ryan, Tobin, and Rollnick [13], SDT can be viewed as a theory that explains the effects that occur when using MI treatments.

Still, there are significant differences in the nature of the two approaches. As pointed out by Vansteenkiste et al. [4], SDT is a macro-theory of human motivation. It began with basic laboratory research and was gradually applied to education [14], to work organizations [15], and to health care [16]. Vansteenkiste et al. thus referred to SDT as a top-down approach to health-behavior change (hypotheses are derived from an overarching theory) [4]. In contrast, the authors spoke of MI as being a bottom-up approach-that is, an approach to health-behavior change that is largely atheoretical and was based on intuition, trial-and-error, and clinical observations when treating addicted patients within the counseling venue.

We agree that SDT was applied in a top-down manner to the health-care domain because at the time that research was begun the theory was quite well developed. At the same time, it is important to recall that the theory's formulation was based on basic research, much of it from the laboratory. We did not start with a theory and then test it. Instead, the initial process of developing the theory involved asking interesting questions and, through research, gradually developing theoretical propositions. As these propositions accumulated, the theory was increasingly applied in the "top-down" manner referred to by Vansteenkiste et al [4]. Nonetheless, throughout its history, the theory has continued to be expanded and refined as new data have shed light on new phenomena and processes.

This is very different from the history of MI, which was developed within the domain of health-behavior change and paid little attention to theory. However, Miller and Rose [17] recently published an article that is aimed toward development of a theory for MI. Its primary focus is on patients' *change talk *as the central mechanism for promoting health-behavior change. Change talk, quite simply, means having patients talk about their behavior change-planning when and how to do it, enumerating the advantages of doing it, guessing how it might affect the people to whom they are closest, and so on. Miller and Rose suggested that the amount of change talk is important, with more being better for yielding change.

We do not disagree that change talk can promote change and be empirically associated with positive outcomes. But fostering change talk of any type seems like a step back from specificity, and may foster controlled as well as autonomous processes. In Miller and Rose's article autonomy seemed implicitly to be somewhat important, but it seemed to have been moved into the background, while the cognitive activity of change talk *per se *became foreground (see [18]). To the degree that this is so-to the degree that the amount of change talk takes a more front-and-center place in the theory than autonomy-the similarity between SDT and MI is diminished. Furthermore, from an SDT perspective, if change talk were to be given prominence, it would be essential that the focus be not just on the *amount *of change talk but rather on the *quality *of the change talk.

More specifically, patients can engage in change talk that reflects autonomy, speaking about such topics as the options available to them, their personal values, and taking greater responsibility because they want to become healthier for themselves and for those who love them. Doing that is likely to be associated with greater internalization, maintained behavior change, and well-being. Alternatively, patients could engage in change talk in a controlled way, emphasizing what they should be doing to change or how their families, friends, or practitioners have been pressuring them to change. Such controlled change talk likely reflects less internalization and will result in less effective and less well-maintained change attempts.

Finally, the fact that there are different qualities of change talk among patients has important implications for practitioners' roles in the process. To the extent that practitioners are directive and controlling in trying to move patients toward a larger quantity of change talk, the results are likely to be less effective. SDT maintains that any such pressure toward a specified outcome will likely foster more interpersonal control, and lower autonomy and relatedness in the interaction. For MI to maintain considerable similarity with SDT it will be necessary, quite simply, for its advocacy of change talk to emphasize practitioners being autonomy supportive and to facilitate patients' engaging in talk about the possibility of autonomous and self-endorsed, rather than heteronomous or externally-driven, change.

## Summary and Conclusions

SDT is a macro-theory of human motivation that has been applied to health-relevant change. Indeed, it may be especially relevant to health care because SDT is centrally concerned with autonomous self-regulation, and autonomy is considered an ethical mandate for medicine. In fact, SDT is highly relevant to health care because it is the only theory that has deeply explored autonomy using empirical methods. In this special issue the results from various randomized trails that have tested SDT were discussed, and the results look promising, providing support for the ideas of practitioners being autonomy supportive and patients becoming more autonomous in their motivation for change. The trials have also been important because they suggest ways to improve future studies.

MI has traditionally been quite congruent with SDT, as both approaches focus on patients' taking responsibility for making important health-related changes. Indeed, some investigators whose work has been discussed in this special issue have indicated that they drew from MI techniques in designing studies to test the SDT model of health-behavior change. Recently, however, Miller and Rose have proposed the initial elements of a theory to explain MI results, which focuses largely on the amount of change talk as the active ingredient for promoting behavior change [17]. This raises some question about the relation of the two approaches, because autonomy seems recently to have been given less importance in MI than was initially the case. Our view is that, for MI and SDT to maintain strong similarity in methods for promoting health-behavior change, the discussions of change talk will need to distinguish between autonomous and controlled change talk and between practitioners being autonomy-supportive rather than controlled in promoting the change talk. We believe that support for autonomy is at the heart of person-centered approaches, including MI, and that it should remain there.
